# Large-Scale Membrane- and Lignin-Modified Adsorbent-Assisted Extraction and Preconcentration of Triazine Analogs and Aflatoxins

**DOI:** 10.3390/ijms18040801

**Published:** 2017-04-11

**Authors:** Shun-Wei Hu, Shushi Chen

**Affiliations:** Department of Applied Chemistry, National Chiayi University, Chiayi 600, Taiwan; s1010331@mail.ncyu.edu.tw

**Keywords:** extraction, preconcentration, lignin-modified adsorbent, adsorption, triazine analog, membrane, aflatoxin

## Abstract

The large-scale simultaneous extraction and concentration of aqueous solutions of triazine analogs, and aflatoxins, through a hydrocarbon-based membrane (e.g., polyethylene, polyethylene/polypropylene copolymer) under ambient temperature and atmospheric pressure is reported. The subsequent adsorption of analyte in the extraction chamber over the lignin-modified silica gel facilitates the process by reducing the operating time. The maximum adsorption capacity values for triazine analogs and aflatoxins are mainly adsorption mechanism-dependent and were calculated to be 0.432 and 0.297 mg/10 mg, respectively. The permeation, and therefore the percentage of analyte extracted, ranges from 1% to almost 100%, and varies among the solvents examined. It is considered to be vapor pressure- and chemical polarity-dependent, and is thus highly affected by the nature and thickness of the membrane, the discrepancy in the solubility values of the analyte between the two liquid phases, and the amount of adsorbent used in the process. A dependence on the size of the analyte was observed in the adsorption capacity measurement, but not in the extraction process. The theoretical interaction simulation and FTIR data show that the planar aflatoxin molecule releases much more energy when facing toward the membrane molecule when approaching it, and the mechanism leading to the adsorption.

## 1. Introduction

Heterocyclic triazine analogs are a crucial precursor for various herbicides [1,2,3]. Among them, chlorine-containing atrazine, propazine, and trietazine are, for the first time, being used on a large scale as derivatizing reagents in acetonitrile for enriching the purity of sulfur-containing acids under alkaline conditions [4]. Melamine is another example of a chemically similar symmetric triazine with numerous common nonherbicide applications [5,6,7,8,9,10,11]. These compounds have either been linked to kidney failure or are considered potential carcinogens and immunotoxins [6,7,8,12,13,14,15,16,17,18]. Previous studies have described numerous gas or liquid chromatography-mass spectrometric approaches for the accurate analysis of triazine isomers and analogs, with or without an enriching medium, to ascertain the level at which they are present in the food chain and the extent of human exposure [18,19,20,21,22,23,24]. The direct removal of only a few triazine analogs in the aqueous environment on an analytical scale, with the assistance of an adsorbent, has been studied [25,26,27,28]. The reported percentage of adsorption was only in the range of 21.9% to 82.9% for the selected triazine analog herbicides.

Agricultural commodities, especially those with high carbohydrate or fat contents, are easily contaminated with aflatoxins under high moisture and high temperature conditions, which favor the growth of the fungal species *Aspergillus flavus* and *Aspergillus parasiticus*. Typical food matrices include maize, cereals, nuts, and soybeans, which are used worldwide, mainly for human consumption [29,30,31]. Many HPLC-oriented methods have been developed for monitoring the food chain for aflatoxin contamination. These methods are usually combined with an extraction process to enrich or recover the analyte before analysis [32,33,34,35,36]. However, reports on the recovery of aflatoxins through adsorption are rare.

Lignin, whose molecular weight can exceed 10,000 daltons, is a biopolymer mixture of three monolignol monomers which have a phenylpropane structure in common. Consequently, it is relatively hydrophobic and aromatic in nature and has a large number of ether linkages and phenyl moieties, yet is unusual because of its heterogeneity and lack of a defined primary structure [37]. Lignin is the most abundant organic material of dead vegetation, is nontoxic and resistant to degradation, and thus, is an extremely versatile material for use in industrial and food processing industries [38,39,40]. However, the application of any lignin-related material for the recovery of molecular pollutants has rarely been seen, except for the metallic Au(III) ion removal from aqueous solution using structurally modified lignin [41,42]. In this study, silica gel modified with a native lignin molecule was used as an adsorbent for facilitating the subsequent process of concentrating an aqueous solution of various triazine analogs and aflatoxins on a large scale, after hydrocarbon-based membrane extraction under ambient temperature and atmospheric pressure. In this study, factors that affect the solvent permeation and the percentage of analyte extracted are discussed, namely the physical properties of the solvent, the nature and the thickness of the membrane, the structure of the analyte, the discrepancy in solubility values of the analyte between the two liquid phases, and the amount of adsorbent used in the process. Finally, the adsorption capacity is measured for several selected analytes, and its dependence on the adsorption mechanism or on the steric hindrance of the analyte is explored through the FTIR approach.

## 2. Results and Discussion

### 2.1. Effect of the Characteristics and Thickness of the Membrane on Permeation

**Figure 1:**
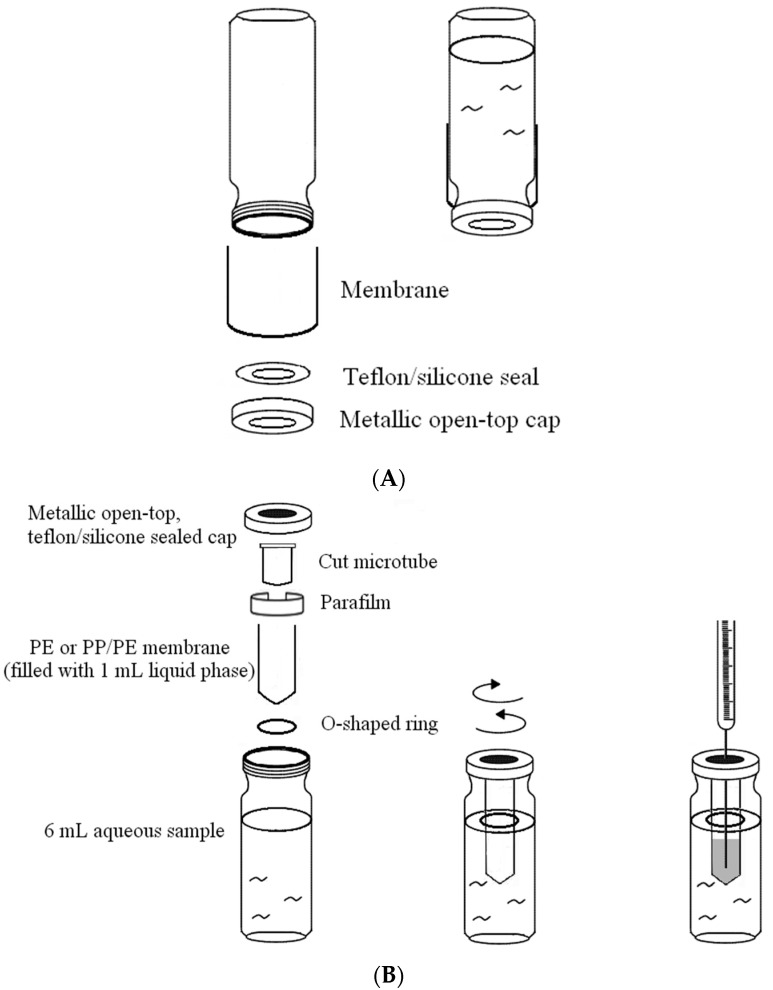
Devices for measuring the permeation of the solvent molecule (**A**) and the percentage of extraction of analytes in hexane and methylene chloride/hexane (**B**).

Microscale membrane extraction has recently become the preferred option for sample preparation in numerous cases because of its simplicity, low operational costs, and high enrichment factors. Polytetrafluoroethylene- and PP-based fiber membranes are two of the most frequently used materials in these studies [43,44,45,46,47,48,49,50,51,52,53,54]. However, the use of flat-sheet membranes constructed of these and other materials in large-scale liquid extraction and concentration under ambient temperature and pressure has not yet been documented. To understand how solvent molecules pass through hydrocarbon-based membranes, and how factors such as vapor pressure, polarity, and the viscosity of the solvent affect the process, the permeation of various solvents under ambient temperature and atmospheric pressure was investigated using the device shown in Figure 1A. The membrane sealed chamber in the container in Figure 1B was designed for the percentage of extraction evaluation.

**Figure 2:**
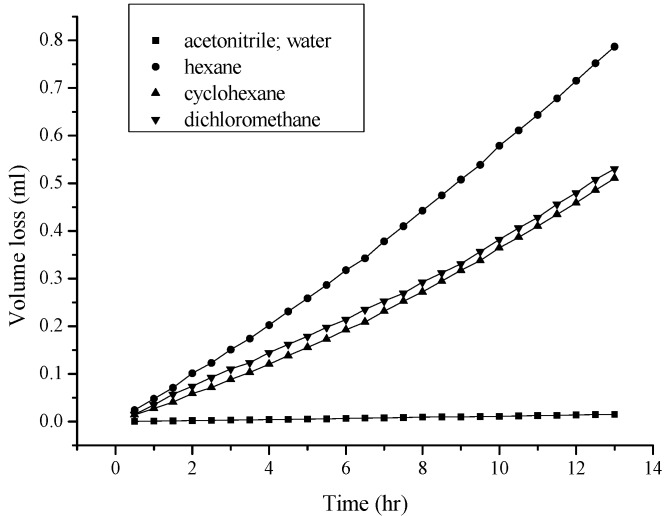
Influence of the nature of the solvent molecule on the permeation through the PE/PP membrane. The thickness of the membrane was 0.010 mm.

**Figure 3:**
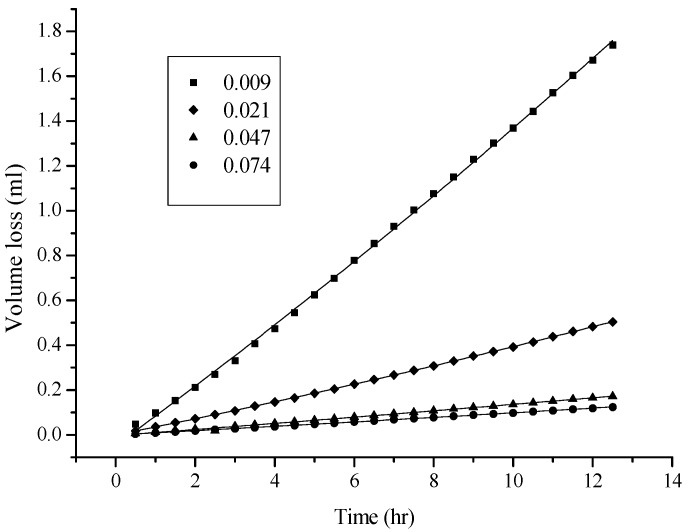
Influence of the thickness (in unit of mm) of the PE membrane on the permeation of the hexane molecule.

**Figure 4:**
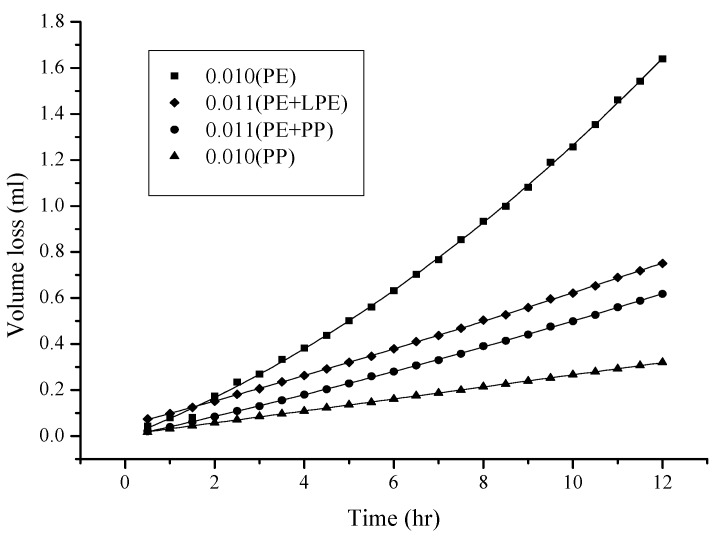
Influence of the nature of the membrane on the permeation of the hexane molecule. The thickness of the membrane was approximately 0.010 mm.

As shown in Figure 2, more volatile, less polar, and less viscous solvents, such as hexane and cyclohexane (see Table 1 for physical properties), permeated through the hydrophobic PE/PP membrane (0.01 mm) more easily. In other words, the hydrophobic membrane was only “wettable” for methylene chloride, hexane, and cyclohexane. Figure 3 shows that the thickness of the membrane (PE) appears to be another factor that affects the permeation of a solvent such as hexane; thicker membranes exhibit lower solvent permeation because of the longer distance to migrate across the membrane. During the process, more time and difficulty would be expected. However, given the thickness of the membrane, the permeation of hexane molecules was further investigated and found to be affected by the nature of the membrane. Among the evaluated membranes, the PE membrane was the most branched, and thus the least dense. The PE/PP membrane was the least branched and the densest, because PP is higher in density, but lower in branching. Consequently, a PE/PP blended membrane should have different porosity properties compared with a PE membrane, as is fully supported by the data in Figure 4. The permeation of hexane molecules was greater for a PE membrane, and lowest for a PP membrane, indicating that a small porous size as a result of high density was an obstacle to the permeation. Despite the magnitude of the permeation, these blended hydrophobic membranes were all wettable to hexane molecules. The coefficient of determination R^2^ for the curves in Figure 2, Figure 3 and Figure 4 was higher than 0.999 in most cases, indicating a satisfactory polynomial fit.

### 2.2. Viscosity and Polarity Effects

Of the examined solvents, water is the most viscous and polar, but the least volatile. Consequently, no permeation for a time period of more than 13 h was observed. Acetonitrile is not as viscous. However, no permeation was measured during the same time period, owing to its high polarity. Conversely, hexane appeared to be the most effective solvent for the permeation process, considering all of the aforementioned properties. Under an aqueous environment, hexane molecules tended to permeate through the hydrophobic membrane and extract the analyte at the interface. Owing to the presence of water molecules, hexane molecules do not permeate further to leave the surface of the membrane because of the incompatibility between the two solvents. In addition, viscous and dense water molecules form a barrier layer to the permeation of hexane molecules at the interface. Thus, water and hexane appear to be an ideal combination in membrane-assisted extraction applications. Acetonitrile is less favorable in that respect. The other practical consideration for a successful extraction process is that the adsorption of triazine analogs on the lignin-modified absorbent was only observed in hexane [55]. The solvent mixture of hexane and methylene chloride (1:1, *v*/*v*) was also used in the evaluation of adsorbing aflatoxins in this study.

### 2.3. Factors Affecting the Extraction Process and Adsorption Capacity Measurement

**Figure 5:**
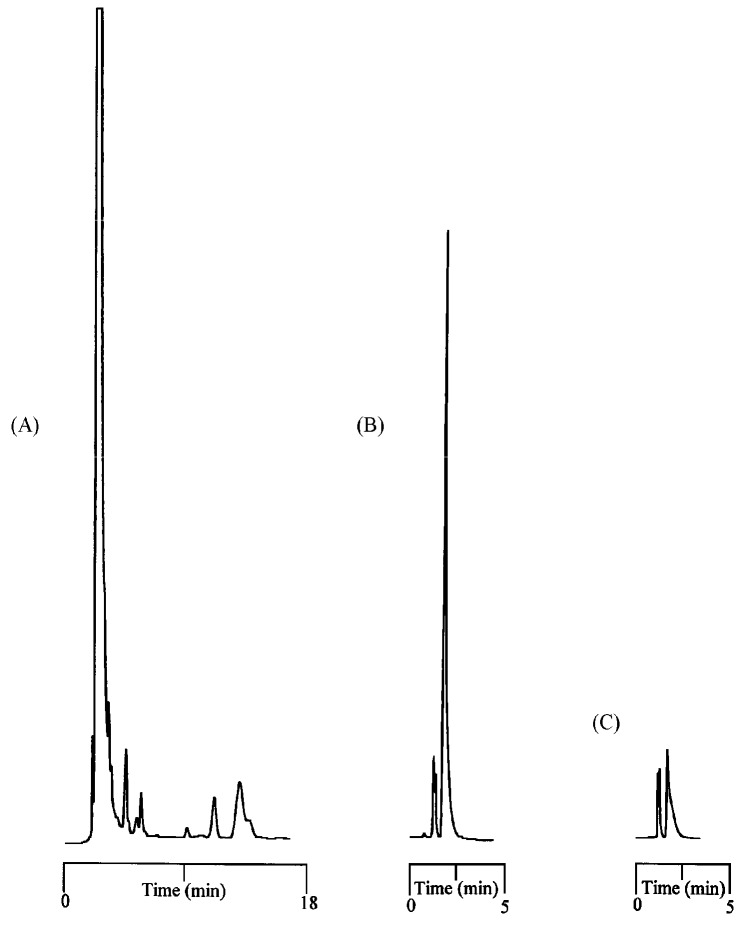
Chromatograms showing the enrichment of ametryne without the presence of an adsorbent in the chamber (**A**) and the residual ametryne outside the extraction chamber (**C**) after a three-day time period, and the standard ametryne solution before the extraction process (**B**) for comparison. A PE membrane was used in the measurement.

**Figure 6:**
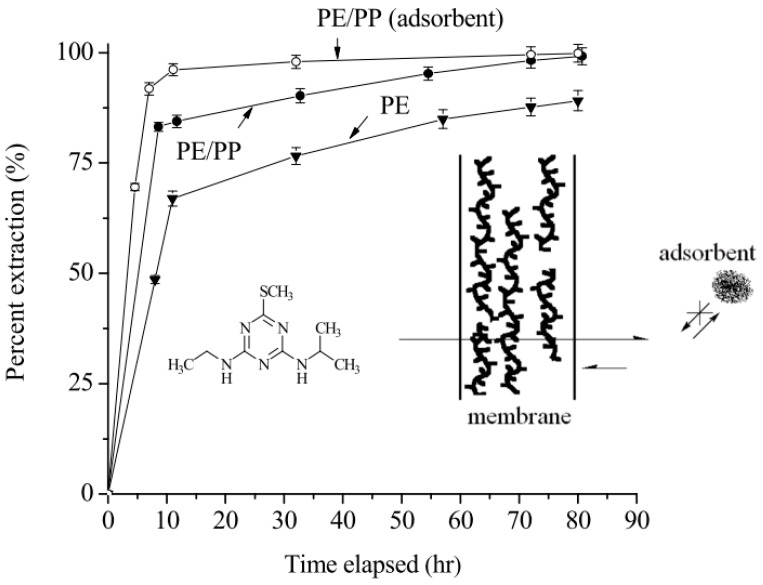
Influence of the nature of the membrane on the percentage of extraction of ametryne in hexane with/without the presence of a lignin-modified adsorbent in the chamber. The thickness of the membrane was about 0.009 (PE), 0.010 (PE/PP) mm respectively. The amount of adsorbent used was 20 mg. Note that the adsorption is irreversible, and the permeation of the analyte molecules in the chamber is counteracted and thus minimized.

The purpose-made device designed for evaluating the percentage of extraction of triazine analogs and aflatoxins is shown in Figure 1B. The membrane, which is sealed with an O-shaped ring, and then a piece of paraffin film, is attached to a support to form the extraction chamber, and is replaceable. A lignin-modified adsorbent can be optionally present in the chamber to facilitate the extraction process. The entire device was sealed, even at the time of sampling for HPLC analysis, to minimize the loss of liquid phase in the chamber. Figure 5 shows the chromatograms for the enrichment of ametryne in the extraction chamber (A) without the presence of an adsorbent, the residual ametryne outside the chamber (C) after a three-day time period, and the standard ametryne solution before the extraction process (B), for comparison under ambient temperature and atmospheric pressure. As can be seen, ametryne was markedly enriched in the extraction chamber. The corresponding percentage of extraction with a PE membrane in this case was calculated to be 87.70%, according to the difference in peak areas. By adding an adsorbent of 20 mg to the extraction chamber, this percentage was reached considerably faster and finally improved to 100% over a 12-h time period. Numerous other triazine analogs examined in this study are listed in Table 2, with data such as the percentage of extraction with or without the presence of the lignin-modified adsorbent in the chamber, and the characteristics of the membrane used in the process. The solubility data for the analyte in both water and hexane at different temperatures are also included for reference and discussion purposes. Upon a close examination of the extraction data in Table 2, a dependence on the characteristics of the membrane was noticed. A more favorable percentage of extraction for a given analyte was always obtained when using the PE/PP membrane. A typical example for the comparison between using the PE and the PE/PP membranes for ametryne extraction without an adsorbent in the chamber is demonstrated in Figure 6. The difference in the percentage of extraction was estimated to be approximately 10% for a three-day extraction in this case. The hydrophobicity, but not the porosity of the membrane, is believed to be one of the factors leading to these results, because a membrane that is relatively hydrophobic should drive the analyte to the surface more easily and efficiently. The analyte near the surface should be further attracted to the liquid phase inside the extraction chamber, as a result of the discrepancy in solubility (see Table 2). However, this driving force was not as effective in the extraction of aflatoxins according to the solubility data shown in Table 3. Note that the percentage of adsorption for aflatoxins was near 100% in a hexane/methylene chloride mixture (1:1, *v*/*v*). Two adsorption mechanisms reported previously were thought to be responsible for the extraction results, and also led to the variation in the adsorption capacity values for these analytes, as shown in Table 3 [55,56]. The availability of binding sites on the lignin-modified adsorbent (i.e., ether linkage in hexane) and the size of the analyte molecule are factors believed to be particularly responsible for the extraction results, according to the proposed structure of lignin [37,42,56,57,58]. However, little evidence was produced that smaller molecules such as triazine analogs were under such a negative influence. Consequently, higher adsorption capacity values were measured because small molecules generated an insignificant steric hindrance during adsorption.

### 2.4. Adsorbent Amount and the Extraction Efficiency

The mass transfer of the analyte in the extraction process was found to be facilitated by placing an adsorbent in the chamber, to irreversibly adsorb the analyte. As shown in Table 2 and Table 3, an improvement in the extraction for several selected analytes was observed. The improvement was enhanced by increasing the amount of adsorbent from 20 to 30 mg. Figure 6 also demonstrates that the extraction percentage for ametryne was accomplished more effectively in a considerably shorter time period by placing an adsorbent in the extraction chamber. The percentage of extraction was improved to nearly 96.2% for a 12-h extraction with 20 mg of adsorbent. A simplified diagram in Figure 6 describes a plausible cause for the extraction improvement resulting from the presence of an adsorbent in the chamber. The irreversible adsorption of the analyte minimized the existing equilibrium between the two liquid phases, thus facilitating the process. This equilibrium could be minimized one step further, or even interrupted, by shifting towards the hexane phase because of almost 100% adsorption, if the major driving force resulting from the discrepancy in the solubility values of the analyte between water and hexane was sufficient. The examples, in Table 2, which include ametryne, prometryne, terbumeton, and dipropetryn, show that the percentage of extraction for a three-day time period reached nearly 100%, which could be accomplished in a shorter time period by adding the lignin-modified adsorbent to the chamber. However, procyazine exhibited the opposite extreme in this particular case, producing nearly no extraction, mostly because of an insufficient discrepancy in the solubility values. The extraction percentage for the analytes in Table 3 was not as marked for the same reason. Interestingly, the extraction process was not noticeably dependent on the size of the analyte. A representative example of this is anilazine, which has three chlorine atoms and one aromatic ring, the percentage of extraction for which reached nearly 85% with the PE/PP membrane, which is higher than that for most of the smaller analytes under the same conditions (see Table 2).

### 2.5. Theoretical Interaction Simulation and FTIR Data

**Figure 7:**
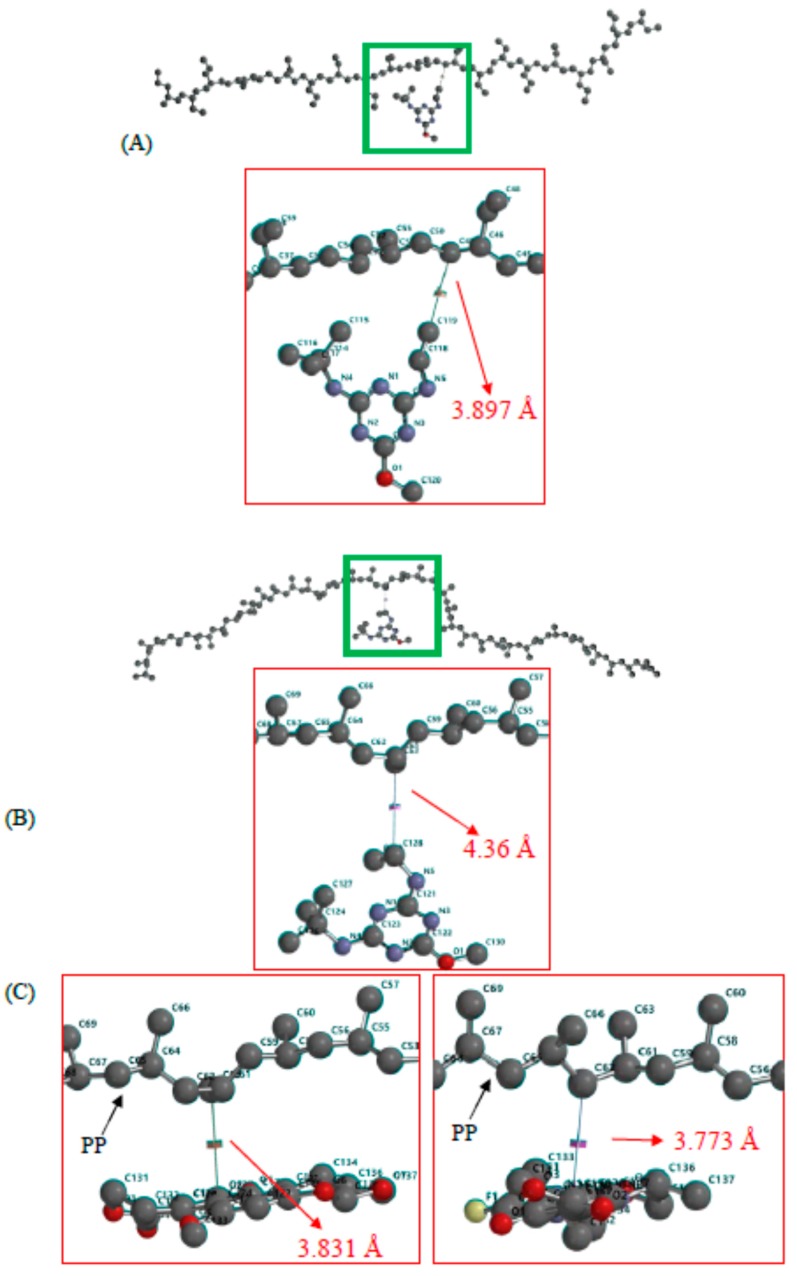
The energy minimization through theoretical computational simulation of the mutual interaction between the membrane (PE: (**A**); PP: (**B**)) and terbumeton molecules (compound **9** in Table 2). A designated area was magnified for a better view. In simulating the interaction between planar aflatoxin G1 ((**C**), left) and PP membrane molecules, only the designated areas were shown after magnification. Note that the aflatoxin G1 molecule is facing toward the membrane segment with a maximum contact area as it approaches it. Antibiotic ofloxacin ((**C**), right), similar in shape to aflatoxin G1, is included for comparison. The red arrows indicate the distance between two designated atoms. Except for the carbon atom in dark grey, oxygen, nitrogen atoms are colored in red, light yellow, respectively. Hydrogen atom is omitted for clarity

These conclusive results and the foregoing discussion are further supported by the theoretical simulation of the single-molecule interaction between PE and PP membranes, and the analyte molecules in Figure 7A,B. More energy (−2379.57 vs. −2253.90 kJ/mol) was released as a terbumeton molecule approached the PP membrane. The oxygen atom (shown in red) in an ether bond was repelled away from the polymer molecule owing to the inconsistency in polarity, and the terbumeton molecule therefore pointed toward the polymer segment from the opposite end. However, in the interaction simulation of planar aflatoxin and antibiotic drug molecules with the PP membrane shown in Figure 7C, the molecules were facing toward the membrane segments, resulting in the release of much higher energies according to the theoretical calculations (−2625.00 and −2840.99 kJ/mol, respectively). Consequently, analytes that are near the surface of the membrane and pointing toward it, but not facing toward it with a maximum contact area like the aflatoxins in Table 3, should more easily, and with a greater likelihood, permeate through the membrane. Subsequently, they should be driven to, and extracted into, the chamber in hexane, because of the discrepancy in the solubility values of the analyte between the two liquid phases. Thus, protonating the analyte to change its characteristics by lowering the pH value of the matrix would be expected to deteriorate the permeability, and thus lower the percentage of extraction (not shown).

**Figure 8:**
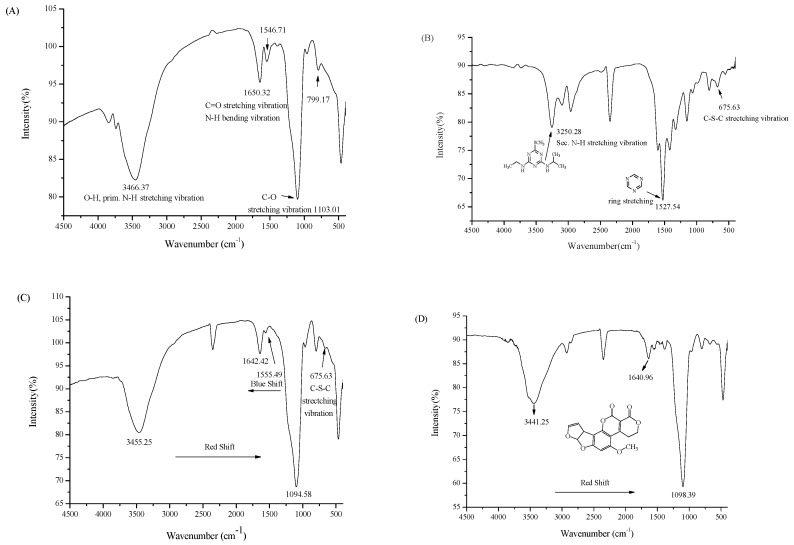
FTIR spectra for lignin-modified adsorbent (**A**), ametryne (**B**), and ametryne/lignin-modified adsorbent (**C**), aflatoxin G1/lignin-modified adsorbent after adsorption (**D**).

However, the FTIR data in Figure 8 for lignin-modified adsorbent (A), ametryne (B), ametryne/lignin-modified adsorbent (C), and aflatoxin G1/lignin-modified adsorbent (D) revealed a significant dipole-dipole oriented interaction that occurred between the analyte and lignin molecules. Upon closely examining these spectra, red-shift measurements were observed for C–O (1103 cm^−1^) and C=O (~1650 cm^−1^) stretching or N–H bending vibrations. In the case of ametryne, data indicated that the sulfur atom, along with the secondary amines in the substituent, were involved in the interaction with the lignin molecule [59]. These secondary amines were the only available sites responsible for the high adsorption with the lignin molecule for triazine analogs containing no sulfur atom. Note that lignin is a supramolecule containing a number of ether linkages for the dipole-dipole interaction with the analyte. Based on the interaction simulation results (not shown), it seemed reasonable enough to consider that one lignin molecule was capable of adsorbing more than one analyte molecule. It also related the lignin-modified adsorbent to the high adsorption capacity and percentage of adsorption in a shorter time period, and to the complexity in the FTIR data after adsorption.

## 3. Experimental Procedures

### 3.1. Apparatus

The HPLC system used in this study was a Hitachi Model L-7100 (Hitachi, Tokyo, Japan), coupled to a D-2500 Chromatopac data station (Shimadzu, Kyoto, Japan), and a UV detector with the detection wavelength set at 260 and 330 nm, for the measurement of triazine analogs and aflatoxins, respectively. A C_18_ column (150 mm × 4.6 mm internal diameter; 5-μm particle diameter) was used for HPLC analysis at a flow rate of 1.0 mL/min in all measurements. The mobile phases for HPLC elution were acetonitrile and a mixture of acetonitrile, methanol, glacial acetic acid, and triethylamine. The FTIR spectra were obtained with a Shimadzu Model FTIR-8400 system. The lignin-modified adsorbent, after the adsorption evaluation, was washed with fresh hexane before being dried and then pelleted with KBr for FTIR measurements. The volume of analyte solution was 100 μL.

Two devices were designed to measure the solvent permeation and the percentage of analyte extracted, as shown in Figure 1. The opening area of the seal perforation for the permeation analysis (top) and the contact area of the membrane for measuring the percentage of extraction (below) were 0.26 and 28.27 cm^2^, respectively.

### 3.2. Chemicals

All chemicals employed in this study, including the lignin (M_n_ = 1750, M_w_ = 14,200), organosilane reagent used as a linker in the chemical immobilization reactions, and the triazine analogs and aflatoxins for the extraction evaluation were purchased from Sigma (St. Louis, MO, USA). The reagents used in the chemical derivatization reactions were purchased from Aldrich Chemical Co. (Milwaukee, WI, USA). The purchased lignin (M_n_ = 1750, M_w_ = 14,200), without further purification, was first immobilized on irregular silica gel (5 μm particle diameter, 100 Å porosity, with a specific surface area of 400 m^2^/g) from Silicycle (Quebec City, QC, Canada), and was then used as the solid phase in the permeation, extraction evaluations, and adsorption capacity measurements at ambient temperatures under atmospheric pressure, according to previously reported chemical derivatization procedures [60,61]. The HPLC grade solvents used to wash the lignin-modified silica gel after preparation and to act as the mobile phase in the HPLC analysis (e.g., toluene, acetonitrile, methanol, triethylamine, methylene chloride, hexane, and ethyl ether), were purchased from Fisher Scientific (Pittsburgh, PA, USA) and Merck Taiwan (Taipei, Taiwan, ROC). In all cases, filtered (0.2 μm) and distilled water was used. Polyethylene (PE), polypropylene (PP), PE/PP, and PE/linear PE membranes with thicknesses of 0.009, 0.010, 0.010, and 0.022 mm, respectively, were obtained from a local supermarket and used without further preparation in this study. Other thicknesses of PE were purchased from Kao-Chia Plastics Co. (Kaohsiung, Taiwan, ROC).

### 3.3. Conditions for Solvent Permeation and Volume Loss Evaluations

To evaluate the solvent permeation, a screw-top vial was filled with 3 mL of solvent and the opening was capped with the membrane, as shown in Figure 1A. The device was placed upside-down during evaluation, to ensure full contact between the solvent and the membrane, and was weighed every half hour to monitor the loss of solvent due to permeation. The weight loss data were then divided by the solvent density to convert the value back to the volume loss.

### 3.4. Conditions for Measuring the Percentage of Extraction of Analyte

A weighed analyte (1 or 0.2 mg) was dissolved in 6 mL of filtered and distilled water, to prepare the solution for measuring the percentage of extraction in hexane. The volume of liquid phase inside the extraction chamber was 1 mL in all cases. In some cases, 10 to 30 mg of lignin-modified adsorbent was added to the extraction chamber to facilitate the process. For a consistent analysis of the results, the membranes examined in the solvent permeation evaluation were also used in the percent extraction study, but with a different device, as shown in Figure 1B. Before the extraction process, the aqueous solution was sampled, and then resampled after a specified period of extraction time under ambient temperature and atmospheric pressure for HPLC analysis. The percentage of extraction was calculated on the basis of the difference in peak areas.

### 3.5. Conditions for Measuring the Percentage of Adsorption and the Adsorption Capacity

A 100-μL quantity of a 2.57 × 10^−3^ M standard solution of selected analyte was added to 10 mg of the lignin-modified adsorbent for a controlled time period. For each measurement, the solution was sampled for HPLC analysis, both before and after the adsorption process, to calculate the percentage of adsorption on the basis of the difference in peak areas. A standard solution was continually added to the matrix in 100 μL increments, until a detectable UV signal was recorded. The HPLC measurement was repeated three times, to obtain an average in all cases. Liquid phases, including hexane and methylene chloride/hexane (1:1, *v*/*v*), were evaluated. The data were then used in the discussion of the extraction and adsorption process.

### 3.6. Theoretical Computational Calculation with Spartan’14 Software

A theoretical calculation for the single point energy was conducted according to a semi-empirical molecular orbital calculation method (Parameterized Model 3), by using Spartan’14 software (Wavefunction, Irvine, CA, USA). The molecular energy was first minimized by modifying the bond lengths and angles until a minimum energy conformer was found prior to the calculation simulation. Atoms on both the membrane and the analyte were simulated to interact with each other, to determine the lowest formation energy at the ground state (i.e., the heats of formation). Segments of PE and PP with molecular weights of 1545 and 1685 amu, respectively, were used in the interaction simulation.

## 4. Conclusions

Emulsion is commonly observed in two or more solvents that are normally immiscible and can be prevented through the use of a membrane. A further application for such an arrangement is to create a confined space in which an adsorbent can simultaneously extract and preconcentrate the analyte. The existing equilibrium of an analyte between two liquid phases can be minimized through irreversible adsorption. This equilibrium can be one step further minimized, or even interrupted, by shifting toward the hexane phase if the discrepancy in the solubility values of the analyte between the water and hexane are marked. Consequently, the extraction process can be facilitated, and thus completed, with a higher percentage of extraction, in a considerably shorter time period. Other factors, such as the nature and the thickness of the membrane, affect the permeation of the solvent molecules and thus the percentage of extraction of the analyte. Notably, the data generated in this study indicate that the influence of the size of the analyte on the percentage of extraction is unclear. However, a steric hindrance effect was observed in the adsorption capacity measurement. Furthermore, the contact area of the analyte permeating the membrane affects the percentage of extraction.

## Figures and Tables

**Table 1:** Some of the physical properties for six selected solvents at 25 °C.

Solvent	Vapor Pressure ^a^ (mmHg)	Dielectric Constant	Water Solubility	Boiling Point (°C)	Viscosity (mPa·s)
Water	23.7	80.10	-	100	0.89
Hexane	151.2	1.88	9.5 mg/L	68.7	0.29
Cyclohexane	97.6	2.02	55 mg/L	80.7	0.90
Ethanol	58.8	24.30	1 kg/L	78.2	1.07
Diethyl ether	532.7	4.33	60.4 g/L	34.6	0.22
Acetonitrile	91.2	37.5	1 kg/L	81.6	0.34
Dichloromethane	352.5	9.1	13 g/L	39	0.43

^a^ Vapor pressure is calculated based on the Antoine equation.

**Table 2:** The percentage of extraction for various triazine analogs with a low-density hydrophobic hydrocarbon-based membrane between the aqueous and the n-hexane phases at room temperature.

No.	Compound ^a^	Analyte Structure	Percent ^b^ Extraction (%)	Percent ^c^ Adsorption (%)	Adsorption ^d^ Capacity (mg)	Solubility ^e^ (H_2_O/HEX)
1	Prometon	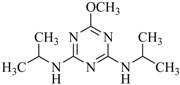	42.23 (1) 41.52 (3) 47.47/77.13 * (2)	~100	-	620/12,000 -
2	Anilazine	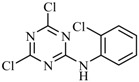	62.13 (1) 55.06 (3) 84.88/~100 * (2)	~100	0.419	8/1700 8/-
3	Ametryne	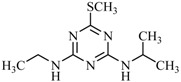	87.70 (1) 90.10 (3) ~100 (2)	~100	0.429	200/14,000 209/-
4	Tebuthiuron *	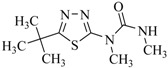	19.66 (1) 27.72 (3) 57.03/86.47 * (2)	~100	-	- 2500/6100
5	Atraton	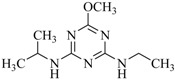	20.97 (1) 28.56 (3) 41.93/67.12 * (2)	~100	-	1800/- -
6	Prometryne	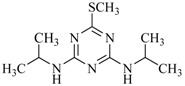	88.60 (1) 98.35 (3) ~100 (2)	~100	0.417	-/5500 33/6300
7	Terbutryne	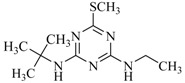	49.36 (1) 86.47 (3) ~100 (2)	~100	0.424	25/9000 -
8	Metribuzin	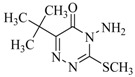	49.14 (1) 51.43 (3) 63.37/89.74 * (2)	~100	-	1050/1000 -
9	Terbumeton	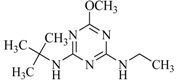	~100 (1) ~100 (3) ~100 (2)	~100	0.432	130/220,000 -
10	Atrazine	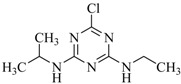	29.59 (1) 7.70 (3) 28.83/55.56 * (2)	~100	-	28/- 33/110
11	Methoprotryne	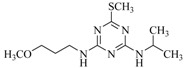	49.09 (1) 59.56 (3) 63.56/91.48 * (2)	~100	-	320/5000 -
12	Dipropetryn	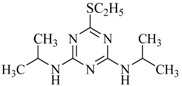	~100 (1) ~100 (3) ~100 (2)	~100	0.426	16/9000 -
13	Simetryn	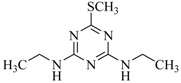	38.04 (1) 42.43 (3) 54.10/79.68 * (2)	~100	-	400/4000 -
14	Procyazine	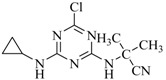	2.33 (1) 1.54 (3) 1.05/- (2)	~100	-	-/50 248/-

^a^ Compound with asterisk (*) is not the atrazine analog; however, it is included for discussion by comparison. ^b^ The analyte is extracted to the hexane phase from the aqueous solution for a three-day time period. The percentage of extraction is calculated based on the difference in peak areas. The volume ratio (H_2_O/hexane) is 6/1. The percent extraction with an adsorbent (20 mg) present in the chamber is marked with asterisk and evaluated over a 12-h time period; however, using the PE/PP membrane only. The mobile phase for HPLC elution is acetonitrile. The type (thickness) of membrane, 1: PE (0.009 mm), 2: PE/PP (0.010 mm), or 3: PE/linear PE (0.022 mm), is expressed in parentheses. According to the information from the providers, PP and linear PE are the minor components in the copolymer products. The linear PE is a four-carbon-based polymer. ^c^ The adsorption evaluation is completed in 1 h in hexane. ^d^ Only available for several selected triazine analogs. ^e^ The solubility data at 20 (above) and 25 °C (below) in unit of mg/L are included for comparison. The acronym HEX stands for hexane.

**Table 3:** The percentage of extraction for aflatoxins with low-density hydrophobic hydrocarbon-based membranes between the aqueous and the organic phases at room temperature.

No.	Compound	Analyte Structure	Percent ^a^ Extraction (%)	Percent ^b^ Adsorption (%)	Adsorption ^c^ Capacity (mg)	Solubility ^d^ H_2_O/Solvent
1	Aflatoxin G1	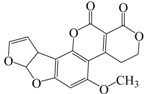	41.76/68.55 (1) 50.14/83.13 (2)	~100	0.290	(10–20) × 10^−3^/5
2	Aflatoxin G2	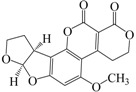	40.76/71.94 (1) 51.13/82.37 (2)	~100	0.293	(10–20) × 10^−3^/5
3	Aflatoxin B1	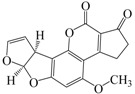	39.56/69.72 (1) 49.82/81.64 (2)	~100	0.297	(10–20) × 10^−3^/10
4	Aflatoxin B2	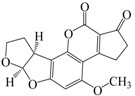	37.56/66.97 (1) 47.03/80.17 (2)	~100	0.295	(10–20) × 10^−3^/5

^a^ The analyte is extracted to the liquid phase from the aqueous phase for a three-day time period. The percentage of extraction is calculated based on the difference in peak areas. The volume ratio (H_2_O/solvent) is 6/1. The percent extraction after the slash is obtained with an adsorbent (30 mg) in the chamber over a 12-h evaluation. Membranes indicated are the followings: 1: PE (0.009 mm), 2: PE/PP (0.010 mm). According to the information provided by the suppliers, PP is the minor component in PE/PP copolymer product. The mobile phase used for HPLC elution is a mixture of acetonitrile, methanol, acetic acid, and triethylamine (475/25/1/2, *v*/*v*). ^b^ The liquid phase used in the evaluation is hexane/methylene chloride (1:1, *v*/*v*). ^c^ The amount of adsorbent used in the measurement is 10 mg. The liquid phase is hexane/methylene chloride (1:1, *v*/*v*). ^d^ The solubility data, in unit of mg/mL, are from chemical suppliers. Aflatoxins are freely soluble in moderately polar solvents, such as chloroform, methanol, and dimethyl sulfoxide, and dissolve in water to the extent of 10–20 mg/L.
